# Massive Hemorrhage Protocol adoption and standardization with a provincial toolkit: a follow-up survey of Ontario hospitals

**DOI:** 10.1007/s43678-025-00929-y

**Published:** 2025-05-22

**Authors:** Chantalle L. Grant, Omar I. Hajjaj, Kimmo Murto, Stephanie Cope, Andrew Petrosoniak, Troy Thompson, Katerina Pavenski, Jeannie L. Callum

**Affiliations:** 1 Department of Surgery, Trauma Services Office, University of British Columbia, Vancouver General Hospital, Vancouver, BC Canada; 2 Postgraduate Program, Department of Surgery, University of Toronto, Toronto, ON Canada; 3 Department of Medicine, Queen’s University, Kingston, ON Canada; 4 Department of Anesthesiology & Pain Medicine, CHEO Research Institute, University of Ottawa, Ottawa, ON Canada; 5 Ontario Regional Blood Coordinating Network (ORBCoN), Toronto, ON Canada; 6 Department of Medicine, University of Toronto, Toronto, ON Canada; 7 Department of Laboratory Medicine and Pathobiology, University of Toronto, Toronto, ON Canada; 8 Department of Pathology and Molecular Medicine, Queen’s University, Kingston, ON Canada

**Keywords:** Massive Hemorrhage Protocol, Trauma, Implementation, Transfusion, Patient Blood Management, Protocole d’hémorragie massive, Traumatisme, Mise en œuvre, Transfusion, Gestion du sang des patients

## Abstract

**Purpose:**

Massive Hemorrhage Protocols improve outcomes for adults with severe hemorrhage, yet only 65% of Ontario hospitals had implemented one by 2018. In response, a Massive Hemorrhage Protocol toolkit was developed and disseminated province-wide in 2021. This study compares Massive Hemorrhage Protocol adoption and content in Ontario hospitals in 2023 versus 2018 using a pre- and post-toolkit rollout survey.

**Methods:**

A 98-question survey was emailed to transfusion medicine laboratory directors or their delegate at 159 hospitals in 2023, 2 years after a provincial Massive Hemorrhage Protocol toolkit rollout that included a 1-day virtual symposium. Results were compared with the 2018 survey containing 82 identical core questions using Chi-square test, Fisher exact test, and Wilcoxon rank-sum nonparametric tests for quantitative data, and content analysis for qualitative data.

**Results:**

The 2023 survey achieved a 100% response rate (*n* = 159); most respondents (*n* = 156) were transfusion staff. Hospitals with a Massive Hemorrhage Protocol increased significantly from 65% (*n* = 150) in 2018 to 77% (*n* = 159) in 2023 (*p* = 0.02). Small transfusion hospitals (< 5000 red blood cell units transfused/year) saw an increase in Massive Hemorrhage Protocol adoption from 60 to 74% (*p* = 0.02). By 2023, 95% (*n* = 159) of hospitals had/were implementing a Massive Hemorrhage Protocol. However, gaps in alignment to evidence-based recommendations remained, including hypothermia monitoring (missing in 25% of Massive Hemorrhage Protocols) tranexamic acid dosing (missing in 19%), and quality metric tracking (missing in 55%). Pediatric content was absent in 45% of Massive Hemorrhage Protocols in health centers caring for children.

**Conclusion:**

The provincial Massive Hemorrhage Protocol toolkit’s dissemination was feasible and associated with increased adoption in Ontario hospitals. Two-years post rollout, 77% of provincial hospitals have Massive Hemorrhage Protocols in place. Opportunities remain to align contents with evidence-based recommendations and expand to remaining hospitals. This strategy could guide other jurisdictions to improve Massive Hemorrhage Protocol adoption and harmonize practices.

**Supplementary Information:**

The online version contains supplementary material available at 10.1007/s43678-025-00929-y.

## Résumé

**Objectif:**

Les protocoles sur les hémorragies massives améliorent les résultats pour les adultes souffrant d’hémorragie grave, mais seulement 65 % des hôpitaux de l’Ontario en avaient mis en œuvre un en 2018. En réponse, une trousse d’outils du Protocole sur les hémorragies massives a été élaborée et diffusée à l’échelle provinciale en 2021. Cette étude compare l’adoption et le contenu du Protocole sur les hémorragies massives dans les hôpitaux de l’Ontario en 2023 par rapport à 2018 au moyen d’une enquête de déploiement préalable et postérieure aux trousses.

**Méthodes:**

Un sondage de 98 questions a été envoyé par courriel aux directeurs des laboratoires de médecine transfusionnelle ou à leur délégué dans 159 hôpitaux en 2023, deux ans après le déploiement d’une trousse provinciale sur le protocole d’hémorragie massive qui comprenait un symposium virtuel d’une journée. Les résultats ont été comparés à l’enquête de 2018 contenant 82 questions de base identiques au moyen du test de Chi-carré, du test exact de Fisher et des tests non paramétriques de somme de rang de Wilcoxon pour les données quantitatives, et de l’analyse de contenu pour les données qualitatives

**Résultats:**

Le sondage de 2023 a obtenu un taux de réponse de 100 % (*n*=159); la plupart des répondants (*n*=156) étaient du personnel de transfusion. Les hôpitaux ayant un protocole d’hémorragie massive ont connu une augmentation significative, passant de 65 % (*n*=150) en 2018 à 77 % (*n*=159) en 2023 (*p*=0,02). Les petits hôpitaux transfusionnels (<5000 unités de globules rouges transfusées/an) ont vu une augmentation de l’adoption du protocole d’hémorragie massive, qui est passé de 60 % à 74 % (*p*=0,02). D’ici 2023, 95 % (*n*=159) des hôpitaux avaient/mettaient en œuvre un protocole sur les hémorragies massives. Toutefois, des lacunes dans l’alignement sur les recommandations fondées sur des données probantes sont demeurées, notamment la surveillance de l’hypothermie (manquante dans 25 % des protocoles d’hémorragie massive) et le suivi des mesures de qualité (manquant dans 55 %). Le contenu pédiatrique était absent dans 45 % des protocoles d’hémorragie massive dans les centres de santé qui s’occupent des enfants..

**Conclusion:**

TLa diffusion de la trousse d’outils du protocole provincial sur les hémorragies massives était réalisable et associée à une adoption accrue dans les hôpitaux ontariens. Deux ans après le déploiement, 77 % des hôpitaux provinciaux ont mis en place des protocoles sur les hémorragies massives. Il reste des possibilités d’harmoniser le contenu avec les recommandations fondées sur des données probantes et de l’étendre aux autres hôpitaux. Cette stratégie pourrait guider d’autres administrations à améliorer l’adoption du Protocole sur les hémorragies massives et à harmoniser les pratiques.

## Clinician’s capsule

**Table:** 

***What is known about the topic?***
Massive Hemorrhage Protocols improve outcomes in hemorrhage, yet in 2018, only 65% of Ontario hospitals had one in place.
***What did this study ask?***
What was the impact of the Ontario provincial Massive Hemorrhage Protocol toolkit on adoption of Massive Hemorrhage Protocols in Ontario?
***What did this study find?***
By 2023, 77% of Ontario hospitals had a Massive Hemorrhage Protocol; an additional 19% were planning to implement a protocol.
***Why does this study matter to clinicians?***
This provincial toolkit model could be utilized to improve uptake and standardization of Massive Hemorrhage Protocols in other jurisdictions

## Introduction

Massive Hemorrhage Protocols and damage control resuscitation were developed to decrease mortality from hemorrhage in adult trauma. Massive Hemorrhage Protocols, in particular, guide overall patient care during life-threatening bleeding by expediting the delivery of pre-defined ratios of blood components, optimizing blood transfusion processes, and mitigating complications related to hemorrhage and/or transfusion [1–4]. Massive Hemorrhage Protocols and damage control resuscitation are associated with improved survival in trauma in adults [1–6].

Despite the benefits of their use, Massive Hemorrhage Protocol implementation has varied globally, ranging from 45 to 100% [7–14]. A Canadian National Advisory Committee recommended all hospitals that transfuse blood should have a protocol in place for massive hemorrhage [15]. However, in Ontario, Canada, our 2018 provincial survey with a 100% response rate (150/150 hospitals) revealed that only 65% of all Ontario hospitals had a protocol in place [16]. In that survey, smaller rural hospitals were less likely to have a protocol; the survey also found tremendous practice variability in activation criteria, laboratory testing, temperature monitoring, and blood pack content; pediatric data were not collected [16].

In response to these findings, the Ontario Regional Blood Coordinating Network (ORBCoN, a provincial Ministry of Health-funded organization) sponsored a multidisciplinary expert consensus group. Over the ensuing 3 years, this group created 42 recommendations and 8 quality metrics for Massive Hemorrhage Protocols, which then informed the development of a consensus and evidence-based Ontario Massive Hemorrhage Protocol (MHP) toolkit for adult and pediatric massive hemorrhage management deployed in April 2021 [17]. The 156-page toolkit included treatment algorithms, simulation material, educational videos, nursing checklists, quality metrics and hand-over tools [18, 19].

Using a pre-test post-test design survey, we compared the adoption, implementation, and content of Massive Hemorrhage Protocols across Ontario before and after the provincial toolkit rollout. We hypothesized that the introduction of the provincial Massive Hemorrhage Protocol toolkit would be associated with higher implementation of Massive Hemorrhage Protocols across the province.

## Methods

### Massive Hemorrhage Protocol toolkit development

Following the results of the pre-test survey in 2018, the toolkit content was developed using a modified Delphi consensus methodology as previously described (three rounds with 36 panelists independently reviewed and reached consensus on 42 statements and 8 quality indicators) [17]. Recommendations from external stakeholders (including pediatric, obstetrics, and resource-limited hospitals) were also incorporated to create a 156-page provincial Massive Hemorrhage Protocol toolkit which was released in 2021. A post-implementation survey, based on the 2018 Ontario survey, was pre-planned to assess Massive Hemorrhage Protocol uptake approximately 2 years after its deployment. Supplementary Fig. 1 depicts the study flow from the initial (2018) to the final (2023) survey.

### Virtual rollout and dissemination

The toolkit was introduced at a virtual online event in April 2021 (Supplementary Fig. 2); the decision to proceed virtually rather than in-person was made due to the impact of the COVID-19 pandemic. All Ontario hospital transfusion medicine laboratory directors received an email invitation, with instructions to disseminate internally. Additional advertising methods included social media, professional development websites, and event branding. Registered attendees were able to submit questions in advance. The event featured 30- to 45-min lectures, followed by moderated question/answer sessions which included pre-and post-lecture knowledge assessments for some sessions. Following the event, the toolkit was made freely available at transfusionontario.org with download tracking. Further dissemination occurred via the ORBCoN monthly newsletter, social media, a podcast, annual hospital site visits, and webinars targeting remote Ontario hospitals from 2021 to 2023.

### Survey development

In 2023, we administered the previously detailed web-based survey to assess Massive Hemorrhage Protocol dissemination, adoption, and implementation, allowing for comparison of Massive Hemorrhage Protocol characteristics to those captured in 2018. The survey (Lime Survey, GmbH, Germany) consisted of 82 common questions from 2018 including short-answer and multiple-choice questions divided into nine categories which captured the “7Ts” of Massive Hemorrhage Protocol characteristics: triggering, team, tranexamic acid, testing, transfuse to target, temperature, and termination [16, 19] (Supplementary Table 1). An additional 16 questions (98 total) were added to capture detailed demographics including pediatric adaptation, alignment with guidelines, and training methods used. As some questions were subject to skip logic, denominators varied according to the question.

### Survey population, administration, and ethics

We invited all 159 hospitals that received blood products from Ontario distribution centers to participate in the 2023 survey, including 158 Ontario hospitals and 1 in Nunavut (the Qikitani hospital site in Nunavut was included as it receives blood products/components and transfusion support from Canadian Blood Services’ production/distribution facility in Ottawa as part of an agreement between the Government of Nunavut and the federal indigenous service department. It is also included in ORBCoN’s annual collaborative site visits with Canadian Blood Services to assess individual hospital utilization and wastage of blood components and products and discuss best practices). The survey was emailed to the medical directors of the Transfusion Medicine Laboratories at each hospital on April 24, 2023, with two follow-up reminders. Recipients were instructed to either complete the survey or forward it to the most responsible person within their hospital for completion. Responses were collected until May 26, 2023. We followed up medical directors from non-respondent hospitals until all completed the survey or confirmed they did not have a Massive Hemorrhage Protocol. The University of Toronto research ethics board reviewed the study and waived ethical review.

### Data analyses

Data was classified and analyzed using descriptive statistics in Microsoft Excel; figures were also created with Microsoft Excel (Microsoft, Redman, Washington, USA). Hospital size was classified by the annual number of transfused red blood cell units reported to Canada Blood Services: < 5000 units (small), 5000–10,000 (medium), and > 10,000 (large). Descriptive statistics were used for quantitative responses. Short answer responses were grouped qualitatively by content analysis. Statistical significance was determined for normal and non-normal distributed data with corrections applied for multiple p value testing.

## Results

There were 342 attendees for the virtual rollout session (with representatives from 92% of hospitals included in the survey [*n* = 159]) including physicians, nurses, medical laboratory technologists, transfusion safety officers, patient blood management coordinators, clinical educators, and administrators. The toolkit content was viewed online over 3500 times and downloaded approximately 1000 times. A total of 477 hospital visits by ORBCoN-associated personnel occurred between April 2021 and March 2023.

### Survey demographics and Massive Hemorrhage Protocol characteristics

The 2023 survey was completed by 156 transfusion medicine laboratory, 1 emergency department, 1 anesthesia, and 1 acute care unit personnel, achieving a 100% response rate (*n* = 159). Similarly, the 2018 survey achieved a 100% response rate (*n* = 150). The number of hospitals varied over time due to closures and the establishment of new facilities throughout the province. Table 1 displays hospital demographics and Massive Hemorrhage Protocol characteristics. The proportion of hospitals with an implemented Massive Hemorrhage Protocol increased significantly (*p* = 0.02) from 65% (*n* = 150) in 2018 to 77% (*n* = 159) in 2023. Most responding hospitals (86%, *n* = 159) were small, and these had a significant (*p* = 0.02) increase in Massive Hemorrhage Protocol adoption, from 60% (*n* = 126) to 74% (*n* = 136) between surveys.

**Table 1:** Hospital demographics and Massive Hemorrhage Protocol (MHP) characteristics

	2018 (%)	2023 (%)	p value^a^
Hospital transfusion activity	*n* = 150	*n* = 159	
Small hospital (< 5000 RBCs/year)	85	86	
Medium hospital (5000–10,000 RBCs/year)	9	11	
Large hospital (> 10,000 RBCs/year)	6	4	
Approved Massive Hemorrhage Protocol at hospital	*n* = 150	*n* = 159	
All hospitals	65	77	**0.020**
Small hospitals **(*n* = 126 in 2018; n = 136 in 2023)**	60	74	**0.018**
Medium hospitals **(*n* = 14 in 2018; *n* = 17 in 2023)**	93	100	0.45
Large hospitals **(*n* = 9 in 2018; *n* = 6 in 2023)**	8	83	0.76
Protocol titles	*n *= 97	*n* = 122	
Massive Hemorrhage Protocol	8	53	** < 0.0001**
Massive Transfusion Protocol	68	11	
Code Transfusion	0	18	
Code Omega	13	12 (10)	
Other	10	7	
Hospital provides care for pediatric patients (< 18)	NA	n = 122	
Yes		83	
No		17	
Massive Hemorrhage Protocol population (provision for pediatric patients)	NA	*n* = 122	
Adults only included in Massive Hemorrhage Protocol		54	
Children only included in Massive Hemorrhage Protocol		0.80	
Adults and children included in Massive Hemorrhage Protocol		45	
Massive Hemorrhage Protocol implemented or updated within the last 5 years	*n* = 97	*n* = 122	
Yes	78	80	0.72

Values in bold are statistically significant (p < 0.05)

^a^*p* value was obtained by Chi-square, Fisher exact, or t test as appropriate; two-sided *p* < 0.05 was considered statistically significant

**Fig. 1:**
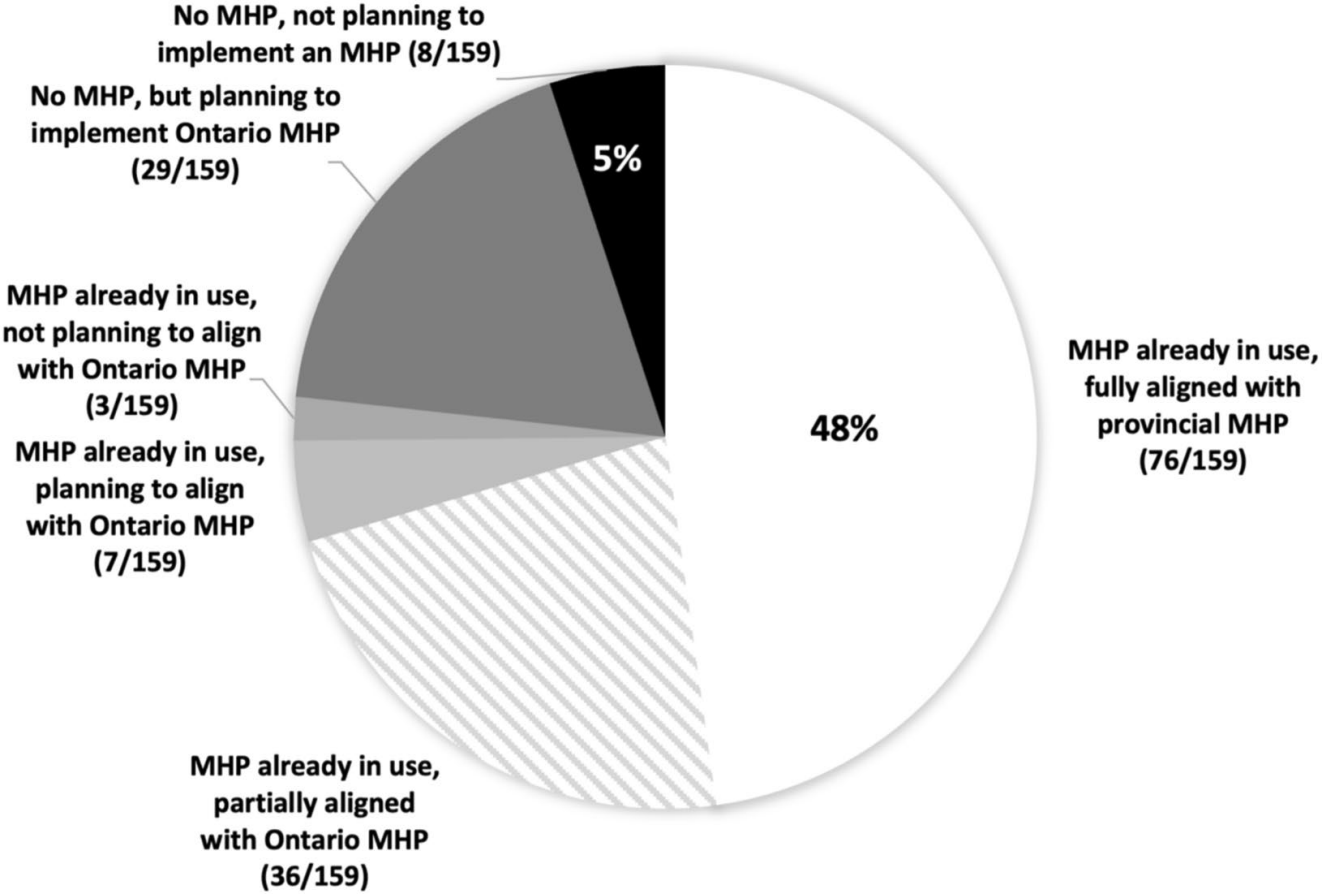
Ontario hospitals’ alignment or planned implementation of the Ontario MHP toolkit (*n* = 159). A total of 95% (151/159) of Ontario hospitals either already have a Massive Hemorrhage Protocol, or are planning on implementing the Ontario provincial Massive Hemorrhage Protocol from the toolkit

Based on the 2023 survey findings, most hospitals (95%, *n* = 159) had already implemented or expressed they were planning to implement a Massive Hemorrhage Protocol, with only eight small hospitals not planning to implement a protocol (5%) (Fig. 1). Of those with an implemented protocol (*n* = 122), 62% were fully compliant with provincial toolkit content and 32% were working toward full toolkit content compliance. Most hospitals (77%; *n* = 122) had recently (< 5 years) implemented or updated their protocols.

*Descriptive caption: A pie chart demonstrating the proportion of Ontario hospitals with or without a Massive Hemorrhage Protocol in use, current alignment with the Ontario MHP toolkit, and intention for implementing the Ontario MHP toolkit. In 48% of* *Ontario hospitals, a Massive Hemorrhage Protocol is already in use and fully aligned with the provincial Massive Hemorrhage Protocol. In 23%, a partially aligned Massive Hemorrhage Protocol is in use, in 4% a Massive Hemorrhage Protocol is in use that plans to align with the Ontario MHP toolkit, and in 2% a Massive Hemorrhage Protocol is in use that is not aligned and not planning to align with the Ontario MHP toolkit. In 18% of hospitals, there is not yet a Massive Hemorrhage Protocol, but plans to implement the Ontario Massive Hemorrhage Protocol from the toolkit. In 5% of hospitals, there is no Massive Hemorrhage Protocol, and no plans to implement the Ontario Massive Hemorrhage Protocol.*

While 83% of hospitals with Massive Hemorrhage Protocols (*n* = 122) provided pediatric care, only 55% of those hospitals had incorporated pediatric content (Table 1).

### Triggers for activation

Although the proportion of Massive Hemorrhage Protocols with activation criteria remained similar in both surveys, there was an increase in the absolute number of protocols with activation criteria in 2023 (79%; *n* = 122) compared to 2018 (85%; *n* = 97). The top three activation criteria were blood loss volume, hemodynamic parameters, and units of red blood cells transfused/anticipated (Table 2).

**Table 2:** Massive Hemorrhage Protocol activation and mobilization

	2018 (%)	2023 (%)	*p*-value^a^
Triggers for protocol activation			
Massive Hemorrhage Protocol with activation criteria	*n* = 97	*n* = 122	
Yes	85	79	0.27
Activation criteria is the same for all patients	*n* = 82	*n* = 94	
Yes	85	74	0.062
Activation criteria	*n* = 82^b^	*n* = 94^b^	
Volume of blood loss	83	68	**0.020**
Hemodynamic parameters (e.g., shock, index, blood pressure, heart rate)	38	64	**0.00060**
Units of red blood cells transfused/anticipated	71	53	**0.016**
Need for uncrossmatched blood	22	21	0.86
No response to crystalloid and bleeding	7	13	0.25
Need for inotropes and bleeding	12	11	0.88
Exclusively at the discretion of the bedside physician	NA	7	NA
Other: use of the ABC score	27	3	** < 0.0001**
Method of activation	*n* = 97^b^	*n* = 122^b^	
Call to locating/security for ‘code’ page	25	71	** < 0.0001**
Call to the transfusion medicine laboratory	78	65	**0.028**
Computerized physician order entry	9	25	**0.0021**
Other (filling out a paper requisition)	21	5	**0.00040**
Team composition, communication, and blood component transport			
Activation rollout team	*n* = 97^b^	*n* = 122^b^	
Physician lead	92	94	0.47
Nursing lead	71	87	**0.0039**
Charting nurse	15	49	** < 0.0001**
Code nurse	15	31	**0.0071**
Anesthesia	14	39	** < 0.0001**
Rapid response team/critical response team	22	36	**0.020**
Porter	64	54	0.14
Transfusion medical laboratory technologist (MLT)	80	91	**0.024**
Coagulation laboratory MLT	19	38	**0.0020**
Hematology laboratory MLT	19	40	**0.00060**
Team member delivering blood products/laboratory samples	*n* = 97^b^	*n* = 122^b^	
Porter	64	60	0.54
Nurse	24	45	**0.0010**
Any available hospital staff	24	19	0.38
Communication of laboratory results to clinical areas	*n* = 97^b^	*n* = 122^b^	
Electronic patient record	62	81	**0.0015**
Phone to local extension	77	61	**0.0086**
Dedicated Massive Hemorrhage Protocol phone	9	4	0.12
Critical laboratory results called	*n* = 97	*n* = 122	
Yes	93	93	0.96
Blood product and component transport containers^c^			
See Supplementary Table 2			

Values in bold are statistically significant (p < 0.05)

^a^*p* value was obtained by Chi-square, Fisher exact, or t test as appropriate; two-sided *p* < 0.05 was considered statistically significant

^b^Respondents could select more than one of the listed options

^c^Some responses were omitted for brevity, see Supplementary Files for the remainder of the responses

### Team composition, communication, and blood component transport

By 2023, an overhead announcement of “Code Transfusion” (71%; *n* = 122) overtook telephone call to the transfusion medicine laboratory (65%; *n* = 122) to communicate activation (Table 2). Team composition primarily included a physician, nurse lead, and a transfusion medical laboratory technician in both years; however in 2023, additional team members were reported. A porter handled most blood and sample transport in both survey years. In 2023, most laboratory results were communicated to clinical areas using an electronic patient record (81%, *n* = 122) and significantly increased from 2018 (*p* = 0.0015). See supplementary Table 2 for transport container utilization.

### Tranexamic acid

Tranexamic acid administration inclusion in the protocol increased in 2023 from 2018 (81% [*n* = 122] vs 70% [*n* = 97]; *p* = 0.06). In 2023, 60% of hospitals with tranexamic acid included in their protocols administered a 2-g dose, compared to only 18% of hospitals in 2018. A 1-g dose of tranexamic acid was specified in 27% of protocols in 2023; details on a second dose of TXA and method of administration were not captured. 

### Temperature management

In 2023, 75% (*n* = 122) of protocols required temperature monitoring, with more sites (78%, [*n* = 91] vs 60% [*n* = 63]; *p* < 0.0001) targeting a higher temperature threshold (> 36°C; Table 3). The most common hypothermia mitigation strategies in both years were warm blankets or forced air blankets, and then rapid infusers.

**Table 3:** Massive Hemorrhage Protocol transfusion, testing and termination

	2018 (%)	2023 (%)	*p*-value^a^
Tranexamic acid			
	*n* = 97	*n* = 122	
Does your protocol use TXA?	70	81	0.056
Dose of TXA administered in protocol	*n* = 66	*n* = 91	
1 g	47	27	
2 g	18	60	
4 g	12	3	
Other or not specified	23	1	
Temperature			
Temperature monitoring	*n* = 97	*n* = 122	
Protocol requires temperature monitoring	63	75	0.12
Temperature targets	*n* = 63	*n* = 91	
> 35 C	25	9	** < 0.0001**
> 36 C	43	74	
> 37 C	17	2	
Other/not specified	14	15	
Mean target temperature (degrees Celsius)	35.7 ± 1.2	35.9 ± 0.35	0.12
Strategies for re-warming available:	*n* = 97^b^	*n* = 122^b^	
Rapid infuser	42	50	0.32
Hotline or blood warmer	28	29	0.79
Warm blankets/forced air blankets	71	70	0.13
Other	4	11	0.081
Bloodwork and Laboratory testing^c^			
See Supplementary Table 3			
Blood product and component transfusion (inventory, packs/boxes, laboratory targets)^c^			
See Supplementary Table 4			
Termination			
MHP with termination criteria	NA	*n* = 122	
Yes		61	
Termination criteria	NA	*n* = 74^b^	
Hemodynamic stability		45	
Hemorrhage control achieved		59	
Futility		31	
Physician discretion		28	
Patient transferred out		28	
Decreased rate of transfusion		26	
Pre-specified laboratory parameters met		5	
MHP provisions and transfer	NA	*n* = 122	
For urgent transfer for definitive management control off site		65	
MHP provisions for patient/SDM notification of activation and potential adverse effects**†**		39	
Education, tracking, and quality improvement			
Education strategies to assist in MHP implementation	NA	*n* = 122^b^	
Email communication to clinical teams		34	
Simulation drills		31	
Mandatory elearning		25	
Rounds, lunch, huddles, learns		20	
Table-top exercise		7	
Quality review	*n* = 97^b^	*n* = 119^b^	
Multidisciplinary debrief/review for any or each MHP	68	67	0.90
Quality metrics tracked for MHP	31	45	**0.030**
Do you perform a review of each MHP?	NA	54	
For which quality metrics are tracked, see Supplementary Table 5			
Which cases have a multidisciplinary debrief?	*n* = 66	*n* = 80	
All cases	41	33	0.29
Select cases based on concern or performance	53	20	** < 0.0001**
Sample cases regardless of concern or performance	2	8	0.13
Other	6	14	0.17

Values in bold are statistically significant (p < 0.05)

^a^*p* value was obtained by Chi-square, Fisher exact, or t test as appropriate; two-sided *p* < 0.05 was considered statistically significant

^b^Respondents could select more than one of the listed options

^c^Some responses were omitted for brevity, see Supplementary Files for the remainder of the responses

### Testing frequency and type of investigations

In 2023, half the hospitals acquired bloodwork at the beginning and end of activation and at predefined intervals (51%, *n* = 122), a significant increase from 2018 (16%, *n* = 97; *p* < 0.0001) (Supplementary Table 3). Similar routine laboratory tests were reported in both surveys; however lactate, blood gas, and ionized calcium measurement increased significantly by 2023. Only four hospitals in both years used rotational thromboelastometry (ROTEM) and none used thromboelastography (TEG).

### Transfusion targets, availability, and administration

Supplementary Table 4 summarizes blood component availability and utilization. Red blood cells were commonly available, and over two-thirds of hospitals had greater than 4 units of frozen plasma available, but less than one-third had available platelets. In the 2023 survey, most hospitals (71%, *n* = 122) reported having provisions to manage patients on anticoagulant/antiplatelet agents. There was a significant increase (*p* = 0.005) in predefined component packs issued during protocol activation, from 61% (*n* = 97) in 2018 to 82% (*n* = 122) in 2023.

The number of hospitals using laboratory-based targets to guide transfusion increased significantly (*p* = 0.0001) from 49% (*n* = 97) in 2018 to 75% (*n* = 122) in 2023. The most common hemoglobin target reported in 2023 was 80g/L (78%, *n* = 122). The adoption of platelet, fibrinogen, and international normalized ratio (INR) targets significantly increased from 47%, 42%, and 44% in 2018 (*n* = 97) to 71% (*p* = 0.0005), 63% (*p* = 0.004), and 67% (*p* = 0.0007) in 2023 (*n* = 122), respectively. Most protocols in 2023 survey favored a restrictive over a liberal target, with protocols targeting platelets at 50 X 10^9^/L (84%), fibrinogen 1.5g/L (81%), and INR 1.8 (78%; *n* = 122).

### Termination

Termination criteria were present in most protocols (61%, *n* = 122) in 2023, commonly defined as hemorrhagic control achieved (59%), hemodynamic stability (45%), and futility (31%, *n* = 74; Table 3). Additionally, most protocols had provisions for urgent off-site transfer for definitive management (65%, *n* = 122).

### Tracking and quality Improvement

The absolute number of hospitals performing a multidisciplinary debrief following protocol activation and termination increased from 2018 (*n* = 97) to 2023 (*n* = 119), although the proportion remained the same (Table 3). There was a significant increase (*p* = 0.03) in sites tracking quality metrics in 2023 (45%, *n* = 119) compared to 2018 (31%, *n* = 97). See Supplementary Table 5 for details on quality metrics.

## Discussion

### Interpretation of findings

The dissemination of the provincial Massive Hemorrhage Protocol toolkit in 2021 was associated with a significant increase in Massive Hemorrhage Protocol adoption across Ontario hospitals, with notable uptake in small hospitals. Most Ontario hospitals had implemented a Massive Hemorrhage Protocol by 2023. Despite this progress, one-quarter of hospitals had plans to implement, but had not yet implemented a protocol by 2023, and eight small hospitals did not even have plans to implement a protocol.

The toolkit provided evidence-based recommendations for Massive Hemorrhage Protocols, and we found a concurrent increase in the uptake of these evidence-based practices after the release of the toolkit, including increased type and frequency of laboratory testing, use of predefined component packs, and restrictive goal-directed transfusion practices. However, gaps in adherence to these recommendations persist, and present opportunities for clinical improvement.

### Comparison to previous studies

The three pillars of patient blood management strategies (diagnosing/treating anemia, minimizing blood loss, and reducing unnecessary transfusions) are all incorporated into the Ontario MHP toolkit [20]. Prior patient blood management initiatives, such as the Ontario Transfusion Coordinator (ONTraC) program, were successfully implemented in Ontario and reduced component use and improved outcomes in joint replacement and cardiac surgery [21]. The Ontario MHP toolkit rollout was similarly successful at implementation leveraging similar educational tactics to the ONTraC program to overcome barriers to program adoption associated with clinician knowledge, attitudes/beliefs, and behaviors [22, 23]. The increasing rate of alignment of hospital protocols with the toolkit’s recommendations and increasing penetration of Massive Hemorrhage Protocols into smaller hospitals suggest the protocol toolkit is acceptable in most hospitals, despite not having embedded hospital champions (e.g., blood transfusion safety officer), hospital contractual obligations, or available outcome data of the ONTRaC initiative [21, 24, 25]. Further, the increased auditing monitoring of quality metrics [26] observed in our 2023 survey suggests efforts to sustain Massive Hemorrhage Protocols after implementation [25, 27].

### Strengths and limitations

The survey results capture Massive Hemorrhage Protocol utilization across Ontario hospitals. This study’s strength lies in its 100% response rate for both surveys, providing a comprehensive view of Massive Hemorrhage Protocol uptake, implementation, and sustainability in Ontario hospitals. The findings also highlight opportunities to standardize protocol content and address disparities. Limitations of our study include its quasi-experimental pre-test post-test design with no ability to control for confounding in this “real-world” research design; for example, we have no information on Massive Hemorrhage Protocol adoption in the years prior to rollout, and reliance on self-reported data from transfusion medicine laboratory personnel may not reflect bedside practices. While our study aligns with implementation science, which promotes evidence-based practice, we were not able to identify barriers to implementation [28–30]. Additionally, we are unable to comment on how Massive Hemorrhage Protocol implementation impacted quality, content compliance, and patient outcomes.

### Clinical Implications

Despite the overall improved adoption of the current Massive Hemorrhage Protocol recommendations, there remain persistent gaps in evidence-based care which present opportunities for clinical improvement. Specifically, temperature monitoring, essential for detecting hypothermia and a critical aspect of damage control resuscitation [18], remains underutilized. Similarly, tranexamic acid administration within 1 h of bleeding onset has not been integrated into all Massive Hemorrhage Protocols, despite being recommended for most cases apart from gastrointestinal hemorrhage [31, 32]. Our findings also suggest that Massive Hemorrhage Protocols are not always adapted to children; this is particularly concerning given that 30-day mortality rates for massive hemorrhage in children are higher than in adults [33, 34]. Addressing pediatric-specific needs in Massive Hemorrhage Protocols is crucial and additional efforts are warranted [35, 36]. Finally, our survey results highlight the controversy that persists regarding the use of viscoelastic testing [37], which remains underutilized in Canada compared to the USA and Europe [38, 39]. The study did not evaluate the use of whole blood, as it had not yet been approved for use outside of clinical studies.

### Research Implications

As of 2023, one-quarter of Ontario hospitals have not yet implemented a Massive Hemorrhage Protocol, highlighting the need for additional research to identify barriers to successful implementation. While the Ontario MHP toolkit includes provisions tailored for hospitals with minimal access to blood components, and the greatest absolute increase in hospital Massive Hemorrhage Protocol adoption occurred in small hospitals, our data still reveal lower rates of protocol implementation in smaller, more rural centers. We hypothesize that staff at these smaller hospitals may perceive Massive Hemorrhage Protocols as unnecessary due to their limited transfusion capacity. Additionally, their sites may face challenges related to insufficient support, funding, and dedicated administrative time for implementation. The lower proportion of hospitals with established Massive Hemorrhage Protocol activation criteria in 2023 could reflect a delay in adoption at smaller hospitals, potentially due to limited familiarity with the latest recommendations or other unknown barriers related to clinician attitudes and behaviors. Furthermore, the timeline for introducing the Ontario MHP toolkit coincided with the height of the COVID-19 pandemic, which likely contributed to delays in implementation. This is reflected by the 29 hospitals without Massive Hemorrhage Protocols that are currently actively working to develop one. While our process utilized implementation science principles [28], it did not apply a formal framework to identify barriers to implementation [29] and this would be the next step for investigation. Strategies to overcome identified barriers could incorporate the European Union’s Patient Blood Management program implementation guidance, which emphasizes creating urgency, empowering staff, and celebrating quick wins, for example, evidence for increased protocol compliance resulting from a “plan–do–study–act” quality improvement cycle [40].

## Conclusions

In summary, this study describes the successful adoption and implementation of a Massive Hemorrhage Protocol toolkit in variable-sized hospitals across a large geographical region. Future studies should identify barriers to Massive Hemorrhage Protocol implementation and strategies to overcome them. A provincial repository of Massive Hemorrhage Protocol activations would allow internal and external benchmarking to improve patient outcomes. 

## Supplementary Information

Below is the link to the electronic supplementary material.

Supplementary file1 (PDF 694 KB)

Supplementary file2 (DOCX 115 KB)

Supplementary file3 (DOCX 60 KB)
