# Supplementary material for: Massive Hemorrhage Protocol adoption and standardization with a provincial toolkit: a follow-up survey of Ontario hospitals
Source: CJEM. 2025 May 22;27(8):614–25. doi: 10.1007/s43678-025-00929-y (PMC12380861; doi:10.1007/s43678-025-00929-y)
Supplement: Supplementary file 1 — Supplementary file1 (PDF 694 KB) [file 43678_2025_929_MOESM1_ESM.pdf]

"Ontario's first recommendations for massive hemorrhage protocol"

(Rescheduled from May 1, 2020 now being delivered on a virtual platform)

April 30, 2021

Speaker	Log In Registration	0645-0700
Dr. Jeannie Callum	Setting the stage for Ontario's MHP	0700-0710
Ms. Kelly Doyle	Patient presentation: The Reason Why We Are All Here	0710-0745
Dr. Allison Collins	Tips for MHP implementation	0745-0755
Dr. Luis Da Luz	Standardized lab tests; tests required and frequency	0755-0805
Dr. Michelle Sholzberg	Blood and products (TXA, Fibrinogen, PCC), substitutions	0805-0825
Speakers	Live Interactive Learning: Q&A	0825-0835
	**Break**	**0835-0845**
Dr. Bourke Tillmann	Temperature /hypothermia	0845-0855
Dr. Jacob Pendergrast	Considerations for small hospitals	0855-0920
Dr. Andrew Beckett	Principals of DCR including activation criteria	0920-0930
Dr. Michelle Zeller	Communication including 'team' components	0930-0945
Dr. Russell MacDonald	Prehospital care - communication and facilitation of early transfer	0945-1000
Speakers	Live Interactive Learning: Q&A	1000-1010
	**Break**	**1010-1020**
Dr. Andrew Petrosoniak	Use of Simulation in massive hemorrhage environment	1020-1040
Dr. Keyvan Karkouti	Cardiac Surgery	1040-1100
Dr. Robert Jee	Obstetrics	1100-1120
Dr. Kimmo Murto	Pediatrics	1120-1140
Dr. Avery Nathens	Quality outcome (tips on monitoring)	1140-1200
Dr. Donnie Arnold	Research opportunities	1200-1210
Speakers	Live Interactive Learning: Q&A	1210-1220
Dr. Katerina Pavenski	Review , next steps and closing remarks	1220-1230
