# Supplementary material for: Massive Hemorrhage Protocol adoption and standardization with a provincial toolkit: a follow-up survey of Ontario hospitals
Source: CJEM. 2025 May 22;27(8):614–25. doi: 10.1007/s43678-025-00929-y (PMC12380861; doi:10.1007/s43678-025-00929-y)
Supplement: Supplementary file 2 — Supplementary file2 (DOCX 115 KB) [file 43678_2025_929_MOESM2_ESM.docx]

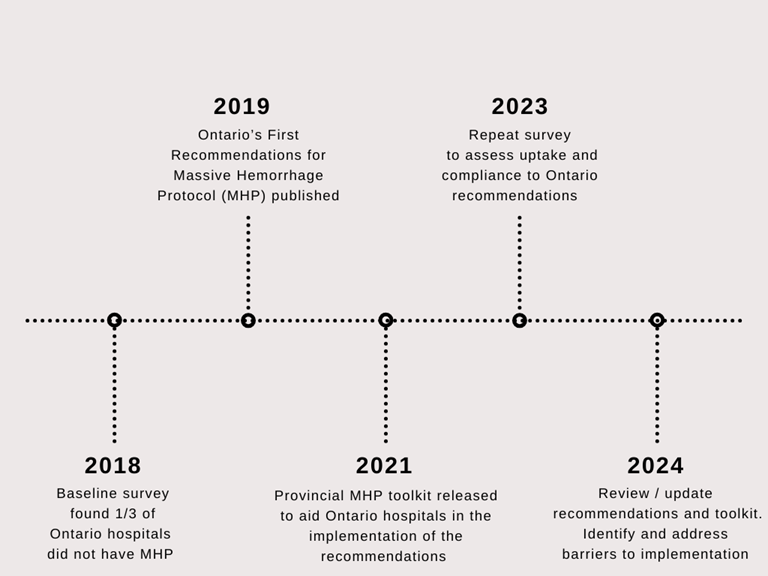


Supplementary Figure 2. Timeline of interventions from initial survey (2018) to development and release of Provincial MHP toolkit (2021) and a repeat survey in 2023. Currently, work is being undertaken to review/update recommendations and toolkit, and identify and address barriers to implementation.
