# Supplementary material for: Massive Hemorrhage Protocol adoption and standardization with a provincial toolkit: a follow-up survey of Ontario hospitals
Source: CJEM. 2025 May 22;27(8):614–25. doi: 10.1007/s43678-025-00929-y (PMC12380861; doi:10.1007/s43678-025-00929-y)
Supplement: Supplementary file 3 — Supplementary file3 (DOCX 60 KB) [file 43678_2025_929_MOESM3_ESM.docx]

**Title: Improving massive hemorrhage protocol adoption and standardization with a provincial toolkit: a follow-up survey of Ontario hospitals**

Authors: **Chantalle L Grant, Omar I Hajjaj, Kimmo Murto, Stephanie Cope, Andrew Petrosoniak, Troy Thompson, Katerina Pavenski, Jeannie L Callum.**

| **Supplementary Table 1.**  Sample of the 82 survey questions. |
| --- |
| **Demographics** |
| What is your primary role? |
| Do you have a hospital approved Massive Hemorrhage Protocol? |
| Are you currently working on an implementation plan to review and align with the Ontario Massive Hemorrhage Protocol where applicable? |
| **Activation Criteria** |
| Do you have activation criteria? |
| Which of the following are used in your activation criteria? |
| How do you activate a Massive Hemorrhage Protocol? |
| Who is included in the activation roll-out as a team member? |
| Who delivers blood/lab samples during a Massive Hemorrhage Protocol? |
| **Communication** |
| How are laboratory results communicated to clinical areas? |
| Are ONLY critical laboratory results called? |
| **Blood Work** |
| How often do you draw a set of blood work during a Massive Hemorrhage Protocol? |
| Which laboratory tests are routinely drawn? |
| **Test Availability** |
| Which test assays are available on site at your hospital? |
| Do you have targeted resuscitation (based on coagulation/platelet counts)? If so, provide target number. |
| What is the total number of lab staff covering all areas at lowest staffing level? |
| **Temperature** |
| Does your protocol require monitoring of patient temperature? |
| What is the temperature target in Degrees Celsius? |
| If the temperature is below target, what strategies are in place? |
| **Transport Containers** |
| When red blood cells, platelets, plasma & cryoprecipitate are required during a Massive Hemorrhage Protocol, are they transported in a validated container?^1^ |
| Which products are routinely stocked on site in the Transfusion Medicine Laboratory? |
| **Transfusion Medicine** |
| Is there a provision in your Massive Hemorrhage Protocol for the management of patients on anticoagulant/ antiplatelet agents? |
| During a Massive Hemorrhage Protocol and when blood group is unknown, what patients receive O-Rhesus (Rh) negative versus O-Rh positive red blood cells? |
| Do you have predefined component packs/boxes that are issued during an Massive Hemorrhage Protocol? What is included in your Massive Hemorrhage Protocol box/pack? |
| **Quality** |
| Do you ever perform multidisciplinary debrief/ review of any Massive Hemorrhage Protocol? |
| Do you track any quality metrics for Massive Hemorrhage Protocol? |
|  |

| **Supplementary Table 2.** Additional questions on containers utilized during Massive Hemorrhage Protocols | | | |
| --- | --- | --- | --- |
|  | **2018 (%)** | **2023 (%)** | **p-value**^‡^ |
| **Product Transport Containers** | **n=97** | **n=122** | **p-value**^‡^ |
| **Red cells (if required) transported in validated container** | **80 (82)** ^+^ | **111 (91)** ^+^ | 0.06 |
| J82 Shipping Container (former CBS boxes) | 47 (48) | 78 (64) | **0.02** |
| Credo shipping container (former Golden Hour) | 5 (5) | 11 (9) | 0.28 |
| Other validated container | 35 (36) | 37 (30) | 0.37 |
| Mobile blood fridge | 0 (0) | 0 (0) | 0.99 |
| **Red cells transported in non-validated container** | **16 (16)** ^+^ | **8 (7)** ^+^ | **0.02** |
| Pneumatic Tubes | 0 (0) | 4 (3) | **<0.01** |
| No container/hand-delivered | 16 (16) | 3 (3) |  |
| Other/not specified | 0 | 1 (1) |  |
| **Platelets (if required) transported in validated container** | **43 (44)** ^+^ | **54 (44)**^+^ | 0.99 |
| J82/E38 shipping container (former CBS boxes) | 22 (22) | 36 (33) | 0.30 |
| Temperature controlled compartment of validated container | 11 (11) | 13 (11) |  |
| Other validated container | 10 (23) | 7 (6) |  |
| **Platelets transported in non-validated container** | **33 (34)** ^+^ | **52 (43)** ^+^ | 0.19 |
| No container/hand-delivered | 17 (18) | 32 (26) | **<0.01** |
| Plastic bag/Other bag | 7 (7) | 15 (12) |  |
| Outside of cooler | 7 (7) | 0 (0) |  |
| Pneumatic tubes | 2 (2) | 4 (3) |  |
| Other/not specified | 0 (0) | 1(1) |  |
| **Plasma (if required) transported in validated container** | **65 (67)** ^+^ | **92 (75)** ^+^ | 0.17 |
| J82/E38 shipping container (former CBS boxes) | 45(46) | 59 (48) | 0.77 |
| Credo Container (former Golden Hour) | 3 (3) | 7 (6) | 0.52 |
| Other validated container | 18 (19) | 33 (27) | 0.14 |
| **Plasma transported in a non-validated container** | **31 (32)** ^+^ | **20 (16)** ^+^ | **<0.01** |
| No container/hand-delivered | 18 (19) | 14 (12) | 0.26 |
| Plastic bag/Other bag | 9 (9) | 2 (2) |  |
| Pneumatic tube | 4 (4) | 3 (2) |  |
| Other container/not specified | 0 (0) | 1 (1) |  |
| **Cryoprecipitate (if required) transported in validated container** | **35 (36)** ^+^ | **21 (17)** ^+^ | **<0.01** |
| J82/E38 shipping container (former CBS boxes) | 18 (19) | 12 (10) | 0.06 |
| Credo (former Golden Hour) | 2 (2) | 2 (2) | 0.82 |
| Other validated container | 13 (13) | 7 (6) | 0.05 |
| **Cryoprecipitate transported in a non-validated container** | **49 (51)** ^+^ | **18 (15)** ^+^ | **<0.01** |
| No container /hand-delivered | 29 (30) | 11 (9) | 0.93 |
| Plastic bag/Other bag | 11 (11) | 5 (4) |  |
| Pneumatic tube | 4 (4) | 1 (1) |  |
| Other container | 5 (5) | 1 (1) |  |

***** Respondents could select more than one of the listed options

^+^*(n for each category calculated by validated containers + nonvalidated containers. Other answers were N/A due to not having that product as part of their Massive Hemorrhage Protocol)*

^‡^ p-value was obtained by Chi-square or Fisher exact test as appropriate, two-sided p<0.05 was considered statistically significant

| **Supplementary Table 3.** Additional Questions on Bloodwork and Laboratory Testing | | |  |
| --- | --- | --- | --- |
|  | **2018 (%)** | **2023 (%)** | **p-value**^‡^ |
| **Timing of bloodwork during an Massive Hemorrhage Protocol** | **n=97*** | **n=122*** |  |
| Beginning and end of activation and at predefined intervals | 16 (16) | 62 (51)) | **<0.01** |
| At the beginning and end of activation | 2 (2) | 6 (5) |  |
| At predefined time intervals (e.g. hourly) | 30 (31) | 24 (19) |  |
| At the start of each pack | 3 (3) | 3 (3) |  |
| At the discretion of the physician | 36 (37) | 16 (13) |  |
| Other | 10 (10) | 11 (9) |  |
| **Duration of pre-defined time intervals** | **n=41*** | **n=86*** |  |
| 30 min | 6 | 3 | **<0.01** |
| 60 min | 29 | 82 |  |
| Other | 6 | 1 |  |
| **Test Assays Available at the Hospital** | **n=97*** | **n=122*** | **p-value**^‡^ |
| Blood gas | 97 (100) | 118 (97) | 0.13 |
| Electrolytes | 97 (100) | 119 (98) | 0.26 |
| Ionized Calcium | 69 (71) | 83 (68) | 0.62 |
| Creatinine | 97 (100) | 120 (98) | 0.50 |
| Calcium | 94 (97) | 118 (97) | 0.94 |
| Glucose | *NA* | 122 (100) | *NA* |
| Magnesium | *NA* | 114 (93) | *NA* |
| Lactate | 95 (98) | 119 (98) | 0.85 |
| International Normalized Ratio (INR) | 96 (99) | 122 (100) | 0.44 |
| Activated Partial Thromboplastin Time (aPTT) | 96 (99) | 122 (100) | 0.44 |
| Fibrinogen | 79 (81) | 95 (78) | 0.52 |
| Complete Blood Count (CBC; including platelet count) | 96 (99) | 122 (100) | 0.44 |
| Group and Screen | 96 (99) | 117 (96) | 0.23 |
| Thromboelastography (TEG) Sample | 2 (2) | 5 (4) | 0.47 |
| Rotational Thromboelastometry (ROTEM) Sample | 9 (9) | 8 (7) | 0.46 |
| **Lab tests Routinely Drawn** | **n=97*** | **n=122*** |  |
| Not specified – physician discretion | 13 (13) | 13 (11) | 0.53 |
| Blood gas | 58 (60) | 100 (82) | **<0.01** |
| Electrolytes | 72 (74) | 101 (83) | 0.12 |
| Creatinine | 60 (62) | 71 (58) | 0.58 |
| Ionized Calcium | 41 (42) | 72 (59) | **0.01** |
| Calcium | 38 (39) | 59 (48) | 0.17 |
| Lactate | 59 (71) | 98 (80) | **<0.01** |
| Glucose | *NA* | 45 (37) | *NA* |
| Magnesium | *NA* | 62 (51) | *NA* |
| INR | 81 (83) | 109 (89) | 0.21 |
| aPTT | 75 (77) | 98 (80) | 0.59 |
| Fibrinogen | 73 (75) | 99 (81) | 0.29 |
| CBC (including platelet count) | 84 (87) | 111 (91) | 0.30 |
| Group and screen | 62 (64) | 90 (74) | 0.12 |
| TEG Sample | 0 (0) | 0 (0) | 0.99 |
| ROTEM Sample | 4 (4) | 4 (3) | 0.73 |
| Other | 20 (21) | 6 (5) | **<0.01** |
| **Healthcare Worker Drawing Blood Samples** | **n=97*** | **n=122*** |  |
| Any trained healthcare worker | 42 (43) | 52 (43) | 0.92 |
| Physician | 22 (23) | 31 (25) | 0.64 |
| Nurse | 51 (53) | 75 (61) | 0.19 |
| Phlebotomy team | 24 (25) | 24 (20) | 0.37 |
| Medical Laboratory Technician (MLT) | 33 (34) | 40 (33) | 0.86 |
| Medical Laboratory Assistant (MLA) | 30 (31) | 59 (48) | **<0.01** |
| Respiratory Therapist (RT), Anesthesia Assistant (AA), Perfusionist | 23 (24) | 21 (17) | 0.23 |
| Other | 6 (6) | 0 (0) | **<0.01** |

*Respondents could select more than one of the listed options

^‡^ p-value was obtained by Chi-square or Fisher exact test as appropriate, two-sided p<0.05 was considered statistically significant

| **Supplementary Table 4. Additional Questions on Blood Components and Products** | | | |
| --- | --- | --- | --- |
|  | **2018 (%)** | **2023 (%)** | **p-value**^‡^ |
| **Products stocked in Transfusion Medicine Laboratory** | **n=150*** | **n=159*** | **p-value**^‡^ |
| Red Blood Cells (all blood groups) | 97 (65) | 91 (57) | 0.18 |
| O Rh-negative RBC | 83 (55) | 107 (67) | **0.03** |
| O Rh-positive RBC | 75 (50) | 102 (64) | **0.01** |
| Platelets 1-2 units | 44 (29) | 40 (25) | 0.41 |
| Platelets 3+ units | 23 (15) | 28 (18) | 0.59 |
| Frozen Plasma 2+ units | 21 (14) | 35 (22) | 0.07 |
| Frozen Plasma 4+ units | 108 (72) | 118 (74) | 0.66 |
| Thawed Plasma | 80 (53) | 10 (6) | **<0.01** |
| Cryoprecipitate 10+ units | 125 (83) | 22 (14) | **<0.01** |
| Prothrombin Complex Concentrate (PCC) (3000 IU+)] | 48 (32) | 142 (89) | **<0.01** |
| Fibrinogen Concentrate (4g+) | 58 (39) | 124 (78) | **<0.01** |
| Recombinant Factor VIIA | 21 (14) | 68 (43) | **<0.01** |
| Pre-labelled trauma stock units | 6 (4) | 29 (18) | **<0.01** |
| **Do you routinely stock thawed Plasma?** | **6 (4)** | **13 (8)** | 0.13 |
| **Hospitals with Routinely stocked blood groups of thawed plasma** | **n=6*** | **n=13*** |  |
| AB | 6 (100) | 8 (62) | 0.13 |
| A | 2 (33) | 4 (31) | 0.91 |
| O | 2 (33) | 2 (15) | 0.56 |
| B | 2 (33) | 1 (8) | 0.22 |
| **Other** | **n=97*** | **n=122*** |  |
| Do you release components to clinical team for transport of patient to another facility? (i.e. not for immediate transfusion)? | 81 (84) | 104 (85) | 0.72 |
| Is there a provision in your Massive Hemorrhage Protocol for the management of patients on anticoagulant/antiplatelet agents? | 57 (59) | 87 (71) | 0.05 |
| Do all patients receive O Rh-negative blood when blood group is unknown? | 31 (32) | 26 (21) | 0.07 |
| During an Massive Hemorrhage Protocol and when blood group is unknown, which patients receive O Rh-negative versus O Rh-positive red blood cells? | n=66* | n=92* |  |
| Biologically female and of childbearing age | 66 (100) | 92 (100) | 0.99 |
| Age 40 or less | 0 (0) | 3 (3) | **<0.01** |
| Age 42 or less | 2 (3) | 0 (0) |  |
| Age 45 or less | 41 (62) | 69 (75) |  |
| Age 50 or less | 17 (26) | 8 (9) |  |
| Other/Not specified | 6 (9) | 12 (13) |  |
| Unknown age or sex get O Rh-negative | 0 (0) | 7 (8) | **0.04** |
| Children (age <16, <18, or unspecified) | 13 (20) | 10 (11) | 0.12 |
| Anti-Rh-D test done first before giving O-Rh positive | 0 | 1 (1) | 0.40 |
| History of Anti-D antibodies | 24 (36) | 14 (15) | **<0.01** |
| **Massive Hemorrhage Protocols with Pre-defined boxes** | **n=97** | **n=122** |  |
| Do you have pre-defined component packs/boxes that are issued? | **59 (61)** | **100 (82)** | **<0.01** |
| Pre-defined packs based on patient weight | *NA* | 19 (16) | *NA* |
| Pre-defined packs based on patient age | *NA* | 81 (66) | *NA* |
| **Pack 1: Units of Red Blood Cells** | **n=58** | **n=99** |  |
| Mean | 4.5 | 3.9 | **<0.01** |
| Standard deviation+ | 1.4 | 0.72 |  |
| Count - 0 units (or not specified)+ | 0 (0) | 0 | 0.99 |
| Count –1 or more units | 58 (100) | 99 (100) |  |
| **Pack 1: Units of Plasma** |  |  |  |
| Mean | 2.3 | 0.44 | **<0.01** |
| Standard deviation | 1.8 | 1.2 |  |
| Count – 0 units (or not specified) | 19 (33) | 86 (87) | **<0.01** |
| Count – 1 or more units | 39 (67) | 13 (13) |  |
| **Pack 1: Units of Platelets** |  |  |  |
| Mean | 0.28 | 0.091 | **<0.01** |
| Standard Deviation | 0.45 | 0.29 |  |
| Count - 0 units | 42 (72) | 90 (91) | **<0.01** |
| Count - 1 or more units | 16 (28) | 9 (9) |  |
| **Pack 1: Units of Cryoprecipitate** |  |  |  |
| Mean | 0.34 | 0 | 0.07 |
| Standard Deviation | 1.8 | 0 |  |
| Count – 0 units | 56 (97) | 99 (100) | 0.14 |
| Count – 1 or more units | 2 (3) | 0 (0) |  |
| **Pack 1: Fibrinogen** |  |  | *NA* |
| Mean | *NA* | 0.36 |  |
| Standard Deviation | *NA* | 1.2 |  |
| Count – No fibrinogen | *NA* | 90 (91) | *NA* |
| Count – fibrinogen included | *NA* | 9 (9) |  |
| **Pack 1: Prothrombin Complex Concentrate** |  |  | *NA* |
| Mean | *NA* | 0 |  |
| Standard Deviation | *NA* | 0 |  |
| Count – 0 units | *NA* | 99 (100) | *NA* |
| Count – 1 or more units | *NA* | 0 (0) |  |
| **Pack 2: Units of Red Blood Cells** |  |  |  |
| Mean | 2.4 | 3.5 | **<0.01** |
| Standard Deviation | 2.0 | 1.2 |  |
| Count - 0 units | 22 (38) | 8 (8) | **<0.01** |
| Count – 1 or more units | 36 (62) | 91 (92) |  |
| **Pack 2: Units of Plasma** |  |  |  |
| Mean | 2.8 | 2.9 | 0.97 |
| Standard Deviation | 1.8 | 1.6 |  |
| Count - 0 units | 12 (21) | 19 (19) | 0.82 |
| Count - 1 or more units | 46 (79) | 80 (81) |  |
| **Pack 2: Units of Platelets** |  |  |  |
| Mean | 0.69 | 0.21 | **<0.01** |
| Standard Deviation | 0.50 | 0.41 |  |
| Count - 0 units | 19 (33) | 78 (79) | **<0.01** |
| Count - 1 or more units | 39 (67) | 21 (21) |  |
| **Pack 2: Units of Cryoprecipitate** |  |  |  |
| Mean | 1.9 | 0 | **<0.01** |
| Standard Deviation | 4.0 | 0 |  |
| 0 units | 47 (81) | 99 (100) | **<0.01** |
| Count – more than 1 units | 11 (19) | 0 (0) |  |
| **Pack 2: Fibrinogen** |  |  | *NA* |
| Mean | *NA* | 0.48 |  |
| Standard Deviation | *NA* | 1.2 |  |
| Count - No fibrinogen | *NA* | 85 (86) | *NA* |
| Count – any fibrinogen | *NA* | 14 (14) |  |
| **Pack 2: Prothrombin Complex Concentrates** |  |  | *NA* |
| Mean | *NA* | 0.36 |  |
| Standard Deviation | *NA* | 1.1 |  |
| Count - 0 units | *NA* | 88 (89) | *NA* |
| Count - 1 or more units | *NA* | 11 (11) |  |
| **Pack 3: Units of Red Blood Cells** |  |  |  |
| Mean | 2.4 | 2.8 | 0.22 |
| Standard Deviation | 2.0 | 1.9 |  |
| Count - 0 units | 22 (38) | 28 (28) | 0.21 |
| Count - 1 or more units | 36 (62) | 71 (72) |  |
| **Pack 3: Units of Plasma** |  |  |  |
| Mean | 2.1 | 1.8 | 0.42 |
| Standard Deviation | 1.9 | 1.4 |  |
| Count - 0 units | 21 (36) | 28 (28) | 0.30 |
| Count - 1 or more unit | 37 (64) | 71 (72) |  |
| **Pack 3: Units of Platelets** |  |  |  |
| Mean | 0.78 | 0.17 | **<0.01** |
| Standard Deviation | 1.1 | 0.38 |  |
| Count - 0 units | 29 (50) | 82 (83) | **<0.01** |
| Count –1 or more units | 29 (50) | 17 (17) |  |
| **Pack 3: Units of Cryoprecipitate** |  |  |  |
| Mean | 0.86 | 0.10 | **<0.01** |
| Standard Deviation | 2.4 | 1.0 |  |
| Count - 0 units | 50 (86) | 98 (99) | **<0.01** |
| Count – 1 or more units | 8 (14) | 1 (1) |  |
| **Pack 3: Fibrinogen** |  |  | *NA* |
| Mean | *NA* | 1.5 |  |
| Standard Deviation | *NA* | 1.9 |  |
| Count – no fibrinogen | *NA* | 61 (62) | *NA* |
| Count – any fibrinogen | *NA* | 38 (38) |  |
| **Pack 3: Prothrombin Complex Concentrates** |  |  | *NA* |
| Mean | *NA* | 0.01 |  |
| Standard Deviation | *NA* | 0.1 |  |
| Count - 0 units | *NA* | 98 (99) | *NA* |
| Count - 1 or more units | *NA* | 1 (1) |  |
| **Pack 4: Units of Red Blood Cells** |  |  |  |
| Mean | 1.8 | 2.1 | 0.69 |
| Standard Deviation | 2.0 | 2.1 |  |
| Count - 0 units | 27 (47) | 48 (48) | 0.81 |
| Count – 1 or more units | 31 (53) | 51 (52) |  |
| **Pack 4: Units of Plasma** |  |  |  |
| Mean | 1.5 | 1.3 | 0.67 |
| Standard Deviation | 1.8 | 1.4 |  |
| Count - 0 units | 27 (47) | 49 (49) | 0.72 |
| Count – 1 or more units | 31 (53) | 50 (51) |  |
| **Pack 4: Units of Platelets** |  |  |  |
| Mean | 0.45 | 0.19 | **<0.01** |
| Standard Deviation | 0.50 | 0.40 |  |
| Count - 0 units | 32 (55) | 80 (81) | **<0.01** |
| Count – 1 or more units | 26 (45) | 19 (19) |  |
| **Pack 4: Units of Cryoprecipitate** |  |  |  |
| Mean | 1.2 | 0 | **<0.01** |
| Standard Deviation | 3.1 | 0 |  |
| Count - 0 units | 50 (86) | 99 (100) | **<0.01** |
| Count – 1 or more units | 8 (14) | 0 (0) |  |
| **Pack 4: Fibrinogen** |  |  | *NA* |
| Mean | *NA* | 0.14 |  |
| Standard Deviation | *NA* | 0.65 |  |
| Count – no fibrinogen | *NA* | 94 (95) | *NA* |
| Count – any fibrinogen | *NA* | 5 (5) |  |
| **Pack 4: Prothrombin Complex Concentrate** |  |  | *NA* |
| Mean | *NA* | 0 |  |
| Standard Deviation | *NA* | 0 |  |
| Count - 0 units | *NA* | 99 (100) | *NA* |
| Count – 1 or more units | *NA* | 0 (0) |  |
| **Pack 5: Units of Red Blood Cells** |  |  |  |
| Mean | 1.5 | 1.3 | 0.48 |
| Standard Deviation | 1.9 | 2.0 |  |
| Count - 0 units | 35 (60) | 66 (67) | 0.42 |
| Count – 1 or more units | 23 (40) | 33 (33) |  |
| **Pack 5: Units of Plasma** |  |  |  |
| Mean | 1.2 | 0.84 | 0.22 |
| Standard Deviation | 1.7 | 1.4 |  |
| Count - 0 units | 35 (60) | 68 (69) | 0.29 |
| Count – 1 or more units | 23 (40) | 31 (31) |  |
| **Pack 5: Units of Platelets** |  |  |  |
| Mean | 0.31 | 0.11 | **<0.01** |
| Standard Deviation | 0.50 | 0.32 |  |
| Count - 0 units | 41 (71) | 88 (89) | **<0.01** |
| Count – 1 or more units | 17 (29) | 11 (11) |  |
| **Pack 5: Units of Cryoprecipitate** |  |  |  |
| Mean | 0.34 | 0 | 0.07 |
| Standard Deviation | 1.8 | 0 |  |
| Count - 0 units | 56 (97) | 99 (100) | 0.14 |
| Count – 1 or more units | 2 (3) | 0 (0) |  |
| **Pack 5: Fibrinogen** |  |  | *NA* |
| Mean | *NA* | 0.3 |  |
| Standard Deviation | *NA* | 1.0 |  |
| Count - No fibrinogen | *NA* | 92 (93) | *NA* |
| Count – any fibrinogen | *NA* | 7 (7) |  |
| **Pack 5: Prothrombin Complex Concentrate** |  |  | *NA* |
| Mean | *NA* | 0 |  |
| Standard Deviation | *NA* | 0 |  |
| Count - 0 units | *NA* | 99 (100) | *NA* |
| Count – 1 or more units | *NA* | 0 (0) |  |
| **Components** | **n=97** | **n=122** |  |
| Is recombinant Factor VIIA a routine part of your Massive Hemorrhage Protocol? | 4 (4) | 9 (7) | 0.39 |
| Does your protocol use fibrinogen concentrate for fibrinogen replacement routinely instead of cryoprecipitate? | 13 (13) | 101 (83) | **<0.01** |
| Does your protocol use PCC routinely instead of plasma? | 14 (14) | 22 (18) | 0.48 |
| **Cryoprecipitate/Fibrinogen concentrate** | **n=97** | **n=119** |  |
| Is cryoprecipitate/fibrinogen concentrate part of your protocol for all Massive Hemorrhage Protocol patients? | 19 (20) | 63 (53) | **<0.01** |
| Is cryoprecipitate/fibrinogen concentrate part of your protocol for obstetric Massive Hemorrhage Protocol patients? | 28 (29) | 27 (23) | 0.30 |
| Is cryoprecipitate/fibrinogen concentrate part of your protocol for cardiac surgery Massive Hemorrhage Protocol patients? | 3 (3) | 2 (2) | 0.66 |
| Is cryoprecipitate/fibrinogen concentrate part of your protocol for non-cardiac surgery patients? | *NA* | 1 (0.84) | *NA* |
| Is cryoprecipitate/fibrinogen concentrate part of your protocol for trauma Massive Hemorrhage Protocol patients? | 1 (1) | 1 (0.84) | 0.88 |
| Is cryoprecipitate/fibrinogen concentrate part of your protocol for Massive Hemorrhage Protocol if fibrinogen below target threshold? | 43 (44) | 29 (24) | **<0.01** |
| Cryoprecipitate/Fibrinogen concentrate not part of protocol | 28 (29) | 15 (13) | **<0.01** |
| Other | 0 (0) | 21 (18) | **<0.01** |
| **Lab-Based Targets for Transfusion** | **n=97** | **n=122** |  |
| Yes | 48 (49) | 91 (75) | **<0.01** |
| No | 49 (51) | 31 (25) |  |
| **Standard Lab-Based Targets for Transfusion** | **n=97** | **n=122** |  |
| **Targets for Hemoglobin as part of Massive Hemorrhage Protocol** | **40 (41)*** | **86 (71)*** | **<0.01** |
| 70 | 26 (65) | 14 (16) | **<0.01** |
| 80 | 5 (13) | 67 (78) |  |
| 90 | 0 (0) | 2 (2) |  |
| Not specified /Other | 7 (18) | 1 (1) |  |
| **Targets for Platelets as part of Massive Hemorrhage Protocol** | **46 (47)** | **86 (71)** | **<0.01** |
| 10 | 0 (0) | 2 (2) | 0.02 |
| 50 | 36 (78) | 72 (84) |  |
| 75 | 0 | 1 (1) |  |
| 100 | 8 (17) | *3(3) |  |
| (Mentioned stipulations for Traumatic Brain Injury requiring platelets >100) | *NA* | *16 (17)* | *NA* |
| **Targets for Fibrinogen as part of Massive Hemorrhage Protocol** | **42 (43)** | **77 (63)** | **<0.01** |
| 1.0 | 13 (13) | 1 (1) | **<0.01** |
| 1.5 | 21 (22) | 62 (81) |  |
| 2.0 or higher | 5 (5) | 7 (9) |  |
| Other | 2 (2) | 5 (6) |  |
| (Mentioned stipulations for obstetrics >2) | *NA* | *27 (35)* | *NA* |
| (Mentioned stipulation for obstetrics >2.5) | *NA* | *2 (2)* | *NA* |
| **Targets for INR** | **43 (44)** | **82 (67)** | **<0.01** |
| 1.2 or 1.3 | 6 (14) | 5 (6) | **<0.01** |
| 1.5 | 19 (44) | 11 (13) |  |
| 1.8 | 9 (21) | 64 (78) |  |
| 2.0 | 2 (47) | 0 (0) |  |
| Other/Not specified | 0 (0) | 2 (2) |  |
| **Targets for aPTT** | **3 (6)** | **0 (0)** | 0.09 |
| 45 | 3 (50) | *NA* |  |
| **Targets for ROTEM/TEG CT** | **1 (2)** | **3 (3)** | 0.63 |
| 80 | 1 (50) | 1 (33) |  |
| 100 | 0 (0) | 2 (67) |  |
| **Targets for ROTEM/TEG A10 MCF** | **1 (2)** | **1 (1)** | 0.87 |
| 7 | 1 (50) | 0 (0) |  |
| 35 | 0 | 1 (100) |  |
| **Targets for ROTEM/TEG A10 FibTem** | **1 (2)** | **n=3 (3)** | 0.63 |
| 7 | 1 (50) | 1 (33) |  |
| 8 | 0 (0) | 2 (67) |  |
| **Targets for ROTEM/TEG ACT** | **0 (0)** | **0 (0)** | 0.99 |
| **Targets for ROTEM/TEG Alpha Angle** | **0 (0)** | **0 (0)** | 0.99 |
| **Targets for ROTEM/TEG MA** | **0 (0)** | **0 (0)** | 0.99 |
| **Laboratory Staffing** | **n=97** | **n=122** |  |
| Presence of an on-call schedule if lab workload exceeds staffing levels | 33 (34) | 49 (40) | 0.35 |
| **Lowest staffing levels** | **n=97** | **n=106** |  |
| Minimum number of transfusion Medical Laboratory Technician staff available | 0.83 | 0.82 | 0.06 |
| Standard deviation | 0.57 | 0.65 |  |
| Minimum number of Core Lab/Hematology/Coagulation Medical Laboratory Technician | 1.11 | 1.21 | 0.29 |
| Standard deviation | 0.86 | 1.0 |  |
| Mean number of Specimen Management Medical Laboratory Technicians | 0.63 | 0.75 | 0.46 |
| Standard deviation | 0.58 | 0.62 |  |

^‡^ p-value was obtained by Wilcoxon rank-sum nonparametric test for lowest staffing levels, and Chi-square or Fisher exact test for other categorical variables, two-sided p<0.05 was considered statistically significant

*Respondents could select more than one of the listed options

| **Supplementary Table 5. Additional Questions on Tracking and Quality Improvement** | | | |
| --- | --- | --- | --- |
|  | **2018 (%)** | **2023 (%)** | **p-value**^‡^ |
| **Quality** | **n=97*** | **n=119*** | **p-value**^‡^ |
| Multidisciplinary debrief/review for any or each Massive Hemorrhage Protocol | 66 (68) | 80 (67) | 0.90 |
| Quality Metrics Tracked for Massive Hemorrhage Protocol | 30 (31) | 54 (45) | **0.03** |
| Do you perform a review of each Massive Hemorrhage Protocol? | *NA* | 65 (54) | *NA* |
| **Which cases have a multidisciplinary debrief?** | **n=66** | **n=80** |  |
| All cases | 27 (41) | 26 (33) | 0.29 |
| Select cases based on concern or performance | 35 (53) | 16 (20) | **<0.01** |
| Sample of cases regardless of concern or performance | 1 (2) | 6 (8) | 0.13 |
| Other | 4 (6) | 11 (14) | 0.17 |
| **Which Quality metrics are tracked?** | **n=30*** | **n=54*** |  |
| Ontario Massive Hemorrhage Protocol Metrics | *NA* | 22 (41) | *NA* |
| % of patients on group-specific blood by pack 2 | 10 (33) | 15 (28) | 0.59 |
| % of patients delivered blood within time frame | 14 (47) | 29 (54) | 0.54 |
| % of patients maintained within hemoglobin within range (60 or 70 to 100) during protocol | 5 (17) | 22 (41) | **0.02** |
| % of patients maintained platelets > 50 during protocol | 5 (17) | 7 (13) | 0.64 |
| % of patients maintained INR | 3 (10) | 10 (19) | 0.36 |
| % of patients maintained fibrinogen >2g/L during protocol | 11 (37) | 5 (9) | **<0.01** |
| % of patients maintained at 36 degrees or higher | 13 (43) | 21 (39) | 0.69 |
| % of activations resulting in blood wastage | 18 (60) | 25 (46) | 0.23 |
| % of patients receiving tranexamic acid | 2 (7) | 24 (44) | **<0.01** |
| % receiving tranexamic acid within a designated time frame of injury (or protocol activation for non-trauma patients | 10 (33) | 20 (37) | 0.73 |
| % of patients with anticoagulants reversed if applicable | 4 (13) | 5 (9) | 0.72 |
| % of Massive Hemorrhage Protocols activated per pre-specified criteria | 16 (53) | 10 (19) | **<0.01** |
| % of Group and screen sent at baseline | 15 (50) | 6 (11) | **<0.01** |
| % of patients with hemorrhage panel (INR, fibrinogen, +/- ROTEM) sent at baseline | 13 (43) | 7 (13) | **<0.01** |
| % of patients with temperature monitored and hypothermia managed if applicable | 11 (37) | 10 (19) | 0.07 |
| % of labs sent q1h per protocol | 7 (23) | 5 (9) | 0.08 |
| % of patients with calcium monitored and corrected if applicable | 7 (23) | 3 (6) | **0.03** |
| % of Massive Hemorrhage Protocols discontinued within 1 hour of last component issued or patient's demise | 14 (47) | 6 (11) | **<0.01** |
| % of Massive Hemorrhage Protocol activations terminated after bleeding was controlled/stopped | 8 (27) | 6 (11) | 0.07 |
| % of complications associated with Massive Hemorrhage Protocol activation | *NA* | 4 (7) | *NA* |
| Other | 13 (43) | 22 (41) | 0.82 |

^‡^ p-value was obtained by Chi-square or Fisher exact test as appropriate, two-sided p<0.05 was considered statistically significant

*Respondents could select more than one of the listed options
